# Plans4Care, a web application providing caregivers personalized solutions to manage dementia-related care challenges: Proof of concept and efficacy trial

**DOI:** 10.1016/j.conctc.2026.101603

**Published:** 2026-01-17

**Authors:** Laura N. Gitlin, Eric Jutkowitz, Catherine Piersol, Sokha Koeuth, Taylor Sivori, Melinda J. Webster, Rachel N. Barnett, David L. Roth

**Affiliations:** a Drexel University, Philadelphia, PA, United States; b Plans4Care, Inc, Providence, RI, United States; c Thomas Jefferson University, Philadelphia, PA, United States; d Johns Hopkins University, Baltimore, MD, United States

**Keywords:** Dementia, Caregiving, Technology, Web application, Care challenges, Personalized solutions

## Abstract

**Objective:**

Few dementia caregivers have access to evidence-based support programs. Web applications (app) may address this gap. Plans4Care, a web app, provides caregivers with on-demand personalized solutions to address care challenges. We present results of a proof-of-concept study and describe a trial protocol to test efficacy.

**Methods:**

To use Plans4Care, caregivers respond to brief onboarding questions, assess dementia patients’ cognitive function, identify care challenges, and generate “action plans” (personalized strategies). Telehealth sessions with dementia-trained care advisors are available. A proof-of-concept study evaluated a clickable prototype using standardized technology scales to determine if >75 % scored positively on four criteria (acceptability, feasibility, appropriateness, ease-of-use). The fully developed app will be tested in a prospective randomized trial (n = 160 caregivers). Caregivers will be assigned to an immediate treatment or 6-month delayed control group to evaluate short (3, 6-months) and long-term (12-month) outcomes on caregiver wellbeing and healthcare utilization of caregivers and people with dementia. App use patterns and care advisor interactions will be evaluated.

**Findings/results:**

Proof-of-concept testing (N = 25 caregivers) resulted in high ratings (100 % achieved for acceptability and feasibility; 80 % for appropriateness; 96 % for usability), supporting full app development. The app contains >100 care challenges, >2700 nonpharmacological strategies, 60+ education-oriented guidance documents, brief how-to videos, novel assessment of cognitive function, an algorithm personalizing strategies to cognitive function and care context, and a care advisor portal. The trial will yield outcome data and utilization patterns to inform commercialization and scaling.

**Conclusions:**

Plans4Care addresses a critical gap in dementia care with potential for commercialization and scalability.

## Highlights

• Plans4Care web app offers tailored, actionable strategies to manage care challenges.
• Web app was developed with iterative input from caregivers and healthcare providers.
• Plans4Care prototype was tested for acceptability, appropriateness, feasibility, and usability.
• Efficacy of Plans4Care will be tested in a randomized wait-list control trial.

## 1 Introduction

Over seven million people in the United States have dementia, with most living at home and cared for by close to 12 million family caregivers [1]. Dementia is characterized by progressive decrements in cognition, function, and behavior such that individuals become increasingly dependent upon family members for daily support [2,3]. As family caregivers assume an increasing number of complex care responsibilities, they face multiple challenges including coordinating care, helping with daily activities of living, assuring home safety and quality of life, managing behavioral and psychological symptoms such as agitation, and/or aggression, planning for long-term care and more, while also taking care of themselves [[4], [5], [6]].

Over the past 50 years, hundreds of caregiver support programs have been developed and tested in randomized trials, with many found to effectively reduce outcomes that matter to caregivers (e.g., depression, burden, distress) and people with dementia (e.g., behavioral symptoms, functional dependence) [7]. However, most efficacious programs are face-to-face or involve online training, time-intensive for caregivers, require training of health professionals for their delivery, and do not easily fit within routine healthcare or community-based organizational workflows. Moreover, most health professionals are unaware of evidence-based programs, nonpharmacological strategies for common dementia-related care challenges, nor how to tailor solutions to care contexts and provide family-centric care. Consequently, the uptake of evidence-based programs has been limited, leaving most families on their own to manage disease progression [[8], [9], [10]].

Digital platforms, web apps, or internet-supported interventions may overcome this research-practice gap by directly providing family caregivers on-demand support and care solutions in real time [11]. These types of interventions have been shown to reduce depressive symptoms and anxiety, enhance caregiver self-efficacy, and afford other caregiver benefits [12]. A randomized trial (n = 100) of a web app providing caregivers generalized knowledge showed a decrease in caregiver depression at 6 weeks [13]. A pilot randomized trial of the WeCareAdvisor, an online platform providing caregivers strategies for managing behavioral symptoms, found that after one month of use, caregivers reported less distress with behaviors and a trend towards reducing the number of behaviors caregivers were actively managing [14].

Recent surveys show that nearly all caregivers are comfortable with computers (97 %), tablets (80 %), and/or cell phones [[15], [16], [17]], with 71 % believing that using web apps can save time, reduce their stress, help their relatives be safer, and increase their own self-efficacy [18]. Moreover, emerging evidence suggests that providing dementia education and strategies to caregivers via web-based formats may improve access to care solutions [[19], [20], [21], [22]].

The proliferation of web apps, online platforms, virtual reality programs, and robotics, along with other related technologies such as Artificial Intelligence (AI), is extremely promising [14]. However, reviews of this burgeoning literature also highlight important concerns and gaps that need to be addressed in future research to optimize the effectiveness and scalability of these approaches [23]. Common limitations include lack of user-centered designs in developing the technology solution, lack of tailoring or individualization in the information and strategies that are provided [24], and focus on only one disease stage, which limits the web app's usability. Tailoring solutions to disease stage and care context is critical when addressing the clinical consequences of dementia, such as functional decline and/or behavioral symptoms that change with the degenerative disease process [25].

In response to the critical public health need to support dementia caregivers, and given the promise of digital platforms, we plan to develop and test a web app, Plans4Care. The Plans4Care web app will provide on-demand, nonpharmacological strategies and educational resources for caregiver-identified challenges that are personalized and tailored to their care context. Plans4Care addresses two types of technologies that are emerging in dementia care: the provision of emotional support and strategies for symptom management [26].

The purpose of this article is to describe the novel features of the Plans4Care web app, present results of a proof-of-concept study (Phase I, Small Business Research Innovation [SBIR] grant funded by the National Institute on Aging), and explain the protocol for a randomized controlled trial that will test the efficacy of the fully developed web app with caregivers (Phase II, SBIR).

Our scientific premise is that providing family caregivers with critical dementia knowledge, support, and validation, combined with actionable strategies to address common care challenges that are personalized to their care contexts, will enhance caregiver wellbeing and ability to manage daily care and reduce healthcare utilization. We hypothesize the mechanistic pathway to be through improving a caregiver's sense of self-efficacy and competence and enhancing their level of readiness to utilize different care strategies (e.g., communicating differently or simplifying tasks).

Plans4Care is a collaborative effort among three partners: the company, Plans4Care, Inc.; Drexel University, which oversees the research evaluations (Phase I and II) of the web app; and Thomas Jefferson University and its physician practice system, which serves as the primary recruitment source for the trial (Phase II).

## 2 Plans4Care web application

The Plans4Care web application (P4C web app) is designed to provide family caregivers with educational materials and personalized action plans on-demand to address caregiver-identified dementia-related care challenges. Caregivers also can meet with a dementia-trained care advisor via telephone/Zoom sessions for additional support, such as guidance regarding implementation of an action plan. The content of the P4C web app has been informed by our over 35 years of National Institute on Aging-funded trials testing face-to-face clinical protocols to enhance the quality of life of family caregivers and people with dementia.

### 2.1 Theories, models, frameworks

The P4C web app is grounded in five theories, models, and frameworks (TMF) described in Table 1. Each of these theories or frameworks has informed the content and approach of the web app.

**Table 1:** Theories, models and frameworks (TMF) informing Plans4Care.

TMF	Brief Description	Application to Plans4Care
Environmental Vulnerability/Reduced Stress-threshold [41]	Model suggests that physical and social environments exert a powerful influence (environmental press) serving as a support or stressor. With disease progression, individuals are increasingly vulnerable to their environment and experience a progressively lowered stress threshold throughout the day.	Strategies provided modify the physical and social environment as it relates to the person with dementia and caregiver to reduce “press” and “vulnerability in the environment.
Cognitive Disabilities Model [42]	The model considers cognitive impairment from a global, multi-component and functional perspective and from which to identify a person's limitations and capacities on everyday functioning.	The Everyday Function Scale is grounded in this model and identifies the cognitive and functional capabilities of a person with dementia are identified to derive the optimal strategy to address a care challenge
Self-determination Theory [43]	Theory suggests that individuals need to be able to make choices and control their daily lives, or otherwise negative consequences are experienced.	Plans4Care strategies help caregivers gain control over day-to-day challenges, achieve care goals, and learn about resources to address unmet needs. It can increase self-determination which can lead to positive mental and physical health.
NIH Stress Health Process Model [44]	The model suggests that objective conditions require caregivers to appraise the demands placed upon them and whether they have the knowledge, skills, and capacity to address objective conditions/care challenges. Caregivers who perceive they are unable to adequately address care demands, perceive their situation as stressful leading to poor mood and other negative sequelae.	Plans4Care provides strategies that help caregivers address objective dementia-related issues (behavioral symptoms), reframe the care context, ways to take care of themselves and disrupt the stress processes caregivers often experience.
Transtheoretical Model of Behavior Change [45]	The model suggests that behavior change is difficult and occurs incrementally through stages.	As care strategies require caregivers to change their behaviors and actions, their level of readiness and willingness to implement strategies are critical to the success of the Action Plan. The number and type of strategies provided is informed by the level of caregiver readiness (based on their responses to brief questions).

In addition to its theoretical foundations, the web app incorporates a usability perspective that prioritizes end user (caregivers, health providers, payers) needs and obtains their feedback throughout the design process in iterative feedback loops to assure app utility for different stakeholders.

To understand the acceptance and use of P4C, we draw upon the Unified Theory of Acceptance and Use of Technology (UTAUT) [27] which identifies four factors influencing tool use: performance expectancy (extent to which individuals believe technology use will benefit them), effort expectancy (ease associated with use of the technology), social influence (extent to which individuals perceive that others believe they should use the technology), and facilitating conditions (belief that there is a supportive infrastructure for use of the technology). UTAUT can inform survey development to capture data that informs marketability and commercialization plans.

Finally, decision-making about web app features has been guided by six core principles shown in Box 1 that serve as the “north star” for P4C development.

Box 1 **Six Core Principles Underlying P4C Web app Development**

• Personalization
• Optimization of choice in engagement with the web app
• Evidence-based content (education materials and strategies)
• Strategies, education materials are wide ranging reflecting relevance to any disease etiology and stage
• Inclusive of common dementia-related challenges (e.g., managing behavioral symptoms and functional decline, safety, and caregiver-centric concerns (stress, need for respite, taking care of self and more)
• Strength-based positive approach (positive affirmations, daily tips, actionable strategies)

Alt-text: Box 1

### 2.2 P4C web app components

P4C has five main complementary and integrated components: the Everyday Function Scale, Care Challenges, Action Plans, Strategies, and a Resource Library (all Company IP, and Provisional Patent).

**Fig. 1:**
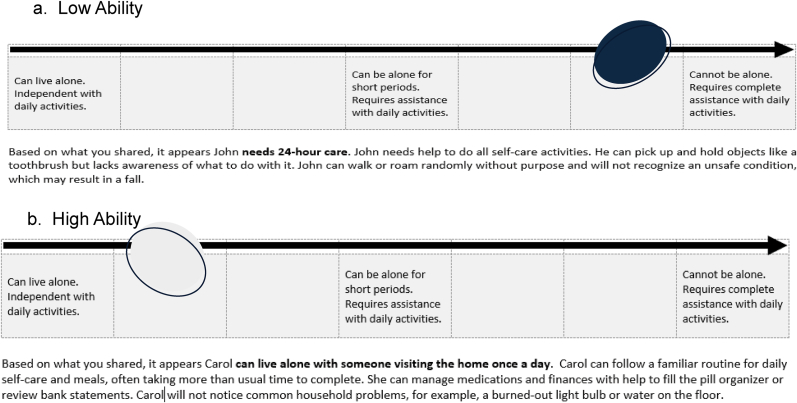
Example of everyday function scale (EFS) Descriptors for high and low functioning.

The Everyday Function Scale. The Everyday Function Scale (EFS) is a novel brief assessment grounded in the Allen Cognitive Disabilities Model [28] and empirical evidence. EFS ascertains the functional capacity of a person living with dementia, which in turn informs the personalization of strategies for managing selected care challenges. Caregivers are presented with four care activities (bathing, dressing, taking medications, using the phone) and asked to choose the one they are most involved with when caring for the person with dementia in the past month. They are first asked to consider which of eight statements (ordered from low to high cognitive functional ability) is “more like” the person with dementia. To determine the EFS rating, caregivers then compare their chosen statement to two others: a statement that reflects a slightly higher level of functioning and a statement that reflects a slightly lower level of functioning to confirm their choice. Once the EFS rating is identified, the web app generates a brief description of what that person can do, including safety considerations (e.g., cannot be left alone at home). Fig. 1 shows examples of descriptions for high and low cognitive functional abilities. The EFS, refined through continuous user inputs from over 15 caregivers and five providers), takes approximately 10 min or less to complete independently by a caregiver.

Care Challenges: The P4C web app addresses over 100 common dementia-related care challenges that are categorized into eight domains (see Table 2). Caregivers select the domain and then the particular care challenge they would like to address within that domain. Challenges are arranged alphabetically within a domain for ease-of-access or a key word search in a search bar can be used. They can focus on any care challenge at any time and generate as many Action Plans as needed. Some care challenges (e.g., wandering) ask caregivers to respond to brief questions (e.g., does wandering occur at night or throughout the day) to generate strategies specific to the care context.

**Table 2:** Eight domains of care challenges.

Domain	Number of Care Challenges^a^	Examples
Person with dementia's selfcare	6	Bathing, dressing, grooming
Person with dementia household tasks	4	Managing finances, light housework, meal preparation
Dementia-related behavioral symptoms	26	Agitation, aggression, rejection of needed help
Person with dementia's mobility	6	Getting in and out of bed
Person with dementia healthcare	9	Hydration, pain, nutrition
Person with dementia leisure	4	Meaningful activities, driving
Person with dementia considerations	9	Home safety, hearing and vision impairments
Caregiver-centered concerns	28	Respite, sleep, taking care of self, care coordination

a Note: Number of care challenges will change as app is further developed.

Action Plans: For each care challenge selected, the app generates an “Action Plan” that is composed of three sections: 1) a summation of the Everyday Function Scale (EFS) as discussed above which provides a brief explanation of the abilities of the person with dementia and potential safety concerns; 2) a brief explanation of why a care challenge may occur (dementia education); and 3) actionable strategies personalized to the person with dementia's cognitive functional abilities and other contextual factors. Caregivers can download, email, and share Action Plans with other family members and/or health providers and generate additional strategies at any time.

Strategies: The Plans4Care database contains over 2700 strategies developed from our previous clinical trials and publications at large. Strategies are categorized into five types described in Table 3 using the bathing care challenge as the exemplar for a person with moderate disease stage. Caregivers initially receive up to three strategies for each of the five categories and can request more strategies for any one category. Caregivers who indicate they are very overwhelmed receive fewer strategies initially (see below).

**Table 3:** Five types of strategies for a bathing challenge for a person with dementia at moderate disease stage.

Strategy Type	Brief Description	General Example	Specific Example	Example of what to Avoid
Effective Communication	Strategies designed to align communicates with cognitive abilities	Hand Sophia each item when needed, and name it as you do.	For example, say, “Here is your razor.”	Avoid giving Sophia multiple items at a time, which can be confusing.
Prompts	Verbal and nonverbal approaches to enhance and guide engagement in a task.	Provide one single-step, simple verbal instruction at a time.	For example, first say, “Brush your teeth,” then say, “Spit in the sink.”	Avoid using long sentences or vague and complex words.
Simplify Environment	Strategies address the physical environment and ways to enhance and guide people with dementia in their home environment	Adjust lighting so it is even throughout the room, without shadows or areas of bright light	–	Avoid letting bathroom light be too dark or uneven, creating shadows that may cause Sophia to become nervous.
Simplify Everyday Activities	Strategies reduce number and complexity of steps to carry out an activity to promote participation.	Allow a good amount of time to complete the activity.	For example, have Sophia complete grooming during the day when others in the household are not waiting for the bathroom.	Avoid scheduling grooming into a tight, pressured time frame, as this may cause frustration for both Sophia and you.
Enhance Engagement	Strategies help people living with dementia engage in valued activities.	Refer to an activity list to help you redirect Sophia to a different activity if Sophia gets agitated or upset.	For example, if Sophia says “No” after you mention brushing their teeth, transition to an unrelated task such as having a cup of tea or listening to music to distract them. Then go back to oral care at a later time.	Avoid rationalizing or arguing with [name] that they must brush their teeth that moment, as this may cause Sophia to get upset and heighten their refusal.

Each strategy is described using a 3-prong approach. Using the care challenge of bathing as an example, a strategy is presented as: 1) a general statement (e.g., “Provide one-step directions”); 2) a personalized example (e.g., First say to [name of person being cared for], ‘Wash your face.’ Then say, ‘Wash your arm”); and 3) what to avoid (e.g, “Avoid using long sentences or vague and complex words, as [name of person being cared for] may not be able to follow what you are saying”).

Strategies are personalized based on several factors. First, caregivers who indicate they are overwhelmed or not ready to try new strategies as part of the onboarding process, initially only receive one or two communication-type strategies. Second, strategies are personalized to the EFS score to obtain the just-right-fit of strategies with a person with dementia's cognitive and functional abilities. Third, the difficulty of implementing strategies (e.g., cost, need for installation, or caregiver time to use) is suggested. Specific dementia care practices are also provided to guide caregivers of people with dementia with other impairments such as vision, hearing, and mobility losses.

Resource Library: The Resource Library offers >60 guidance documents covering a range of topics relevant to dementia caregiving at any stage of the disease process such as home safety considerations or planning for the future and possible living considerations. These documents complement Action Plans by providing in-depth information about a particular topic of relevance to dementia care. The Resource Library also contains brief (<2 min) instructional and engaging videos that illustrate best dementia care practices such as how to use redirection, relax the rules, declutter, implement activities that match interests and abilities, and more. Caregivers can browse the Resource Library when they choose and download, save, or share documents.

### 2.3 Other features

The P4C web app contains other features designed to provide emotional support, validation, and enhance dementia knowledge. Caregivers can rate their stress level as frequently as they choose and track their ratings over time. Furthermore, caregivers receive positive affirmations through weekly tips on the dashboard. Finally, caregivers can choose to schedule a video or audio session with a care advisor if their particular care challenge is not represented, if they have difficulty implementing the Action Plan, they want to discuss a care concern, or as stated above, they are very overwhelmed and unsure how to manage. Care advisors, who may have any health professional background, are trained in a caregiver-centric approach including how to build rapport, support caregivers and meet them where they are at, problem solve and establish actionable goals and strategies via teleconferencing.

### 2.4 How caregivers will use the P4C web app

**Fig. 2:**
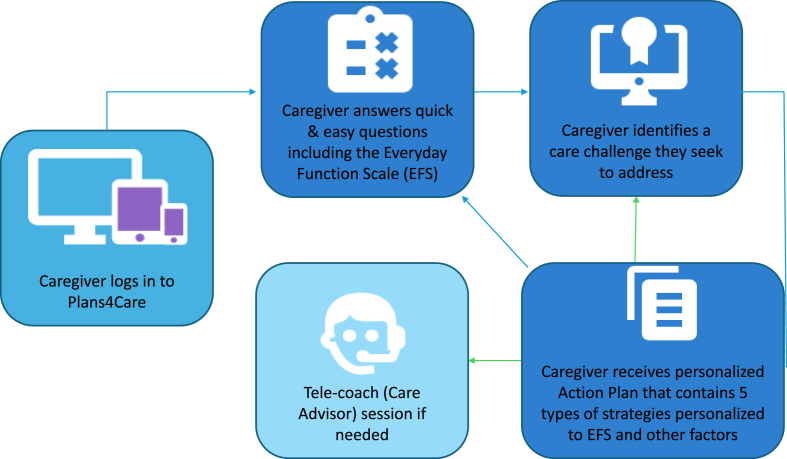
Plans4Care flow for caregivers.

Fig. 2 depicts how caregivers will use the web app to generate personalized Action Plans. Consider a daughter caring for her father with dementia. She receives a link from her dad's primary care provider, installs the application, and the software guides her through brief, easy-to-answer onboarding questions (Company IP) about herself and her father and she completes the EFS. She browses the Resource Library for dementia information specific to her concern about her father's home safety. She prints out the guidance document to share with her family. She then seeks to address wandering outside the home as her top initial concern. She answers a few questions specific to her father trying to leave home and then generates an Action Plan. The software uses a novel clinical algorithm (Company IP) on the back end and maps her responses to her father's cognitive functional capacity (EFS rating) to evidence-based strategies in which, for this particular care challenge, there are 62 unique strategies. Her personalized Action Plan contains a description of the cognitive functional capacity of her father, a brief explanation of why dementia-related wandering may occur, and specific strategies reflecting the five areas specified above and which follow the 3-prong format (Table 3). Examples of strategies in this case might include: environmental modifications (alarm by door); introducing an activity matched to her father's interests/abilities (sorting coins as he was a former accountant) prior to the time of day he tends to want to leave home; and simple statements to redirect her father (“let's get a snack”), resolving the care challenge. She also seeks tele-support with a care advisor to practice communicating more effectively and using other strategies on her Action Plan to address other care challenges. She schedules a session via the web app's calendar, receives a notification of the appointment, and connects with the care advisor through the app which uses secure, HIPAA compliant software.

Whereas other marketed digital caregiver programs offer general information/tips, our solution personalizes Action Plans to address challenges of most concern to families and along the factors described above. Personalization is key to the effective use of nonpharmacological strategies and efficacious web apps [29,30].

#### 2.4.1 Engagement indicators

Engagement is a critical mechanism by which apps may have benefits for its users. We will enhance engagement by providing caregivers with a brief initial training in the use of the app and its features along with setting up an introductory session with a care advisor. We have define a priori the dose, exposure and engagement as follows:

Exposure/Access: Caregivers in the immediate treatment group can use P4C for up to 12 months and those randomized to delayed access can use the app for 6 months. This amount of exposure is considered best practice and enables caregivers to address multiple changing care challenges as dementia progresses.

Time-based Dose: As caregivers use the app on-demand based on their needs, we anticipate variable use rates. However, engagement level will be evaluated based on these criteria:

#### 2.4.2 Minimal engagement involves:

1. Caregiver completes brief training in use of app by trainer;
2. Caregiver completes brief onboarding questions including the Everyday Function Scale.

#### 2.4.3 Moderate engagement:

#1 and 2 above.

3. Caregiver logs in at least once a month over a 6-month period.
4. Caregiver uses at least 2 core features of the app over a 6-month period such as:
  • Obtain new strategies for existing care challenges
  • Identify new care challenge
  • Reassess person with dementia using EFS scale
  • Schedule session with a care advisor
  • View Resource Library (educational guidance documents or brief instructional videos)
  • Rate stress levels

#### 2.4.4 High engagement:

#1, 2, 3 and 4.

5. Caregiver completes 3 full cycles using the app which includes: identifying at least 3 care challenges, generating associated action plans, indicating which strategies on action plans they intend to try.

## 3 Proof-of-concept study (Phase I)

To establish proof-of-concept, we developed a clickable prototype and systematically tested it with 25 caregivers. As a low-risk study, this was an expedited review from Drexel University's Institutional Review Board (Protocol number: 2306009970A001).

The study was designed in two parts: an unmoderated activity in which caregivers tried the app on their own, and a moderated group discussion, in which caregivers shared their experiences using the app. The study was designed to address four quantitative milestones identified a priori which served as our peer-reviewed and NIH approved “go/no-go” criteria for determining whether full development of the web application was warranted. These criteria included: 1) ability to enroll 25 caregivers in prototype testing; 2) ≥75 % of enrolled caregivers completed three assigned tasks independently (onboard, identify one of five care challenges, and generate an action plan) to evaluate feasibility; 3) ≥75 % of enrolled caregivers rated the prototype as acceptable using three standard measures (described below); and 4) ≥75 % rated navigation easy-to-use (score ≥68 on the Systems Utility Scale-SUS, described below). The prototype addressed five common dementia-related care challenges: bathing (resistance to, rejection of needed help, or physical difficulties bathing); dressing (resistance to, rejection of needed help or physical difficulties dressing); wandering inside the home (pacing, walking aimlessly); trying to leave home; and anxiety.

### 3.1 Recruitment and eligibility

Family caregivers were recruited through User Interviews, a company providing access to potential research participants across the United States who volunteer to test technologies (https://userinterviews.com). Eligible caregivers were either a family member, friend, or neighbor who self-identified as caring for a person with dementia of any etiology or disease stage; were ≥21 years of age; English speaking; and actively (past 6 months) managing or interested in learning how to manage one of the five areas stated above. Finally, caregivers had to be willing to participate in an unmoderated test (e.g., using the prototype on their own), and then a moderated portion that entailed participating in a 1-h focus group using Zoom.

Interested caregivers who responded to the User Interviews call for research participation responded to a brief set of questions reflecting eligibility criteria on the User Interviews platform. Within two days, 163 individuals responded to the call for study participation, of whom 60 were eligible. Through the User Interviews platform, eligible caregivers received a link to e-consent and Non-disclosure Agreement (NDA) forms embedded in Drexel's REDCap and were asked to review and check agreement to study participation and the NDA. Upon receipt of agreement to these two forms, caregivers were sent a link through the User Interviews platform to the Plans4Care prototype and instructions for onboarding. This resulted in a sample of 25 heterogeneous (race, ethnicity, relationship to the person with dementia, age, geographic location) caregivers of people with dementia who were recruited and enrolled within a two-week time frame.

### 3.2 Test procedures

The proof-of-concept evaluation involved two components: (1) an unmoderated (self-directed) use of the clickable prototype and response to survey items to determine if four milestones were achieved; and (2) a moderated portion consisting of a 1-h focus group to provide qualitative data regarding the user experience and obtain input to inform future web app development. The moderated component did not inform milestone achievements but rather was designed to provide insights for product development.

Unmoderated Use of Clickable Prototype: Upon signing consent and non-disclosure agreement (NDA) forms, caregivers were given five days to access a link to the clickable prototype and complete three tasks independently: (1) answer brief onboarding questions, (e.g., relationship to person for whom they care; (2) select one of five care challenges of interest and respond to a few additional brief questions specific to the care challenge that facilitate further tailoring of strategies (e.g., for bathing, “do you have a grab bar?”), and (3) click a button to generate and review an Action Plan that describes why the particular challenge may occur and specific nonpharmacologic strategies tailored to the care situation to address it. Following these three steps, participants then clicked a link in the prototype that led them to Drexel's REDCap system in which they were asked to complete on their own brief (anonymous, de-identified) survey questions (22 items) to assess acceptability, usability, and ease of navigation of the clickable prototype. The scores of these measures informed whether the a priori identified milestones (go/no-go criteria) were achieved.

Measures: Caregivers assessed acceptability using three brief validated scales each of which has 4-items for a total of 12 items: Acceptability of Intervention Measure (AIM; e.g., “Plans4Care is appealing to me”); Intervention Appropriateness Measure (IAM; e.g., “Plans4Care seems applicable”); and Feasibility of Intervention Measure (FIM; e.g., “Plans4Care seems easy to use”) [31,32]. These three measures are indicators of the extent to which end-users believe an intervention is acceptable, appropriate, and feasible. Caregivers scored each item along one of five responses (1 = completely disagree to 5 = completely agree).

Caregivers also completed the Systems Usability Scale (SUS), an industry standard 10-item and easy-to-use survey that is a validated scale to assess usability of patient systems/apps [33,34]. SUS can be used with small sample sizes with reliable results, effectively differentiating between useable and unusable online systems. Caregivers scored 10 items along one of five responses (1 = strongly disagree to 5 = strongly agree) with each item assessing perceived ease of navigation (“I thought the system was easy to use”), if technical support is needed (“I think that I would need the support of a technical person”), and utility (“I think that I would like to use this system frequently”).

Moderated Focus Groups: Following completion of the unmoderated portion of the evaluation, caregivers participated in one 60-min focus group conducted by the Drexel University research team. Each focus group consisted of 4–6 caregivers, was conducted via Zoom and recorded, and was moderated by two Drexel University research staff members with technical assistance provided by the Company's technology partner, OCTO. Each focus group was structured similarly: (1) brief welcoming remarks and introductions (10 min); (2) review of participants' unmoderated experiences with the platform and their satisfaction with and ease using the platform (10 min); (3) relevance and perceived utility of the Action Plan and its strategies (10 min); (4) review of other features to consider for building the web app (e.g., resource library topics, brief how to videos), in addition to discussing facilitators and barriers to using a web app as part of care routines (10 min); and (5) impression of its look and feel (20 min). Following completion of five focus groups of 5 caregivers, transcripts were systematically scrubbed for identifiable information (e.g., caregiver names); themes were identified using the qualitative software, MAXQDA [35], for each focus group question; and a codebook was generated. A member of the research team then independently reviewed transcripts using the codebook. Finally, the research team met to identify themes, inconsistencies in the codebook, and finalize the analysis. While not part of the calculations for milestone achievements, the thematic analysis provided valuable feedback for the further development of the web app.

### 3.3 Survey results

Table 4 summarizes the results organized by milestone for the Phase I protocol testing phase.

**Table 4:** Prototype milestones (go/no-go criteria), measurement and achievement.

Criteria	Domain Assessed	Measurement	How Calculated	% Achieved
**N=25** enroll and complete study (unmoderated and moderated focus group sessions)	Acceptability	# Enrolled	# completing unmoderated use of clickable prototype and attending moderated focus group	**100 % achieved.**25 caregivers completed unmoderated and moderated sessions.
**≥75 %** complete 3 tasks (onboard, identify 1 care challenge, generate action plan)	Feasibility	Complete clicks on platform	% completing all 3 tasks	**100 % achieved.**25 caregivers completed all three tasks on the prototype.
**≥75 %** rate Plans4Care acceptable	Acceptable, appropriate, appealing	3 measures (AIM, FIM, IAM) each have 4 items with 5 response categories (total k = 12)	% scoring between 48 (agree) to 60 (completely agree)	**80 % achieved.**Caregivers scored between agree to completely agree on these measures [20 of 25 caregivers scored between 48 and 60].
**≥75 %** rate navigation easy to use (score ≥68)	Usability	Systems Usability Scale (10-items, 5 response categories)	1. Recode 2. Add items together 3. Multiply by 2.5 to convert scores of 0–40 to 0-100 4. Calculate % with score≥ 68	**96 % achieved.**Caregivers scored the prototype platform easy-to-use [24 of 25 caregivers scored ≥68].

Milestone #1 (Enroll 25 caregivers who complete unmoderated and moderated sessions). We enrolled a highly heterogeneous sample of 25 family caregivers with regard to gender, age, ethnicity, race, relationship to and living arrangement with the person with dementia, years caregiving, and geographic location. As shown in Table 5, of the 25 enrolled caregivers, 11 (44 %) identified as male and 14 (56 %) identified as female. Caregivers were on average 44 (SD = 14.13) years of age with a range of 22–75 years old. For ethnicity and race, 1 (4 %) identified as Hispanic; 24 (96 %) were non-Hispanic. Close to half identified as white/Caucasian (n = 12, 48 %) with the other half identifying as Black/African American (n = 8, 32 %), Asian (n = 4, 16 %), or multi-racial (n = 1, 4 %). Also, 16 (64 %) study participants lived with people with dementia whereas 9 (36 %) did not. The average time spent caregiving was 3.39 (SD = 1.89) years. As to geographic regions (as defined by the Census Bureau's four statistical regions), 7 participants lived in the Northeast, 7 in the South, 7 in the West, and 4 were from the Midwest. A total of 12 states were represented. As to the caregiver's relationship to the person with dementia, 2 (8 %) were spouses, 13 (52 %) were adult children, 9 (36 %) were grandchildren, and 1 (4 %) was a great-grandchild. For gender of the person living with dementia, 8 (32 %) identified as male, and 17 (68 %) identified as female. The enrolled caregivers may have had experience with web apps as they tended to be young, 36 % were grandchildren, and they were all registered on a recruitment platform that specializes in technology testing.

**Table 5:** Background characteristics of sample (N = 25).

	Total Sample (n = 25)
**Caregiver Characteristics**	
Gender (number, %)	
Male	11 (44 %)
Female	14 (56 %)
Age	
Mean (SD)	44 (14.13)
Range	22–75
Ethnicity (number, %)	
Hispanic	1 (4 %)
Non-Hispanic	24 (96 %)
Race (number, %)	
White/Caucasian	12 (48 %)
Black, African American	8 (32 %)
Native American or Alaskan Native	0 (0 %)
Asian	4 (16 %)
Native Hawaiian or Pacific Islander	0 (0 %)
Multi-racial	1 (4 %)
Live with PWD (number, %)	
Yes	16 (64 %)
No	9 (36 %)
Time caregiving in Years (M ± SD)	3.39 ± 1.89
Geographic location (number, %)	
Northeast (New Jersey, New York, Pennsylvania)	7 (28 %)
South (Florida, Georgia, Maryland, North Carolina, South Carolina, Texas)	7 (28 %)
Midwest (Illinois, Indiana, Wisconsin)	4 (16 %)
West (California)	7 (28 %)
Caregiver relationship to PWD (number, %)	
Spouse	2 (8 %)
Adult Child	13 (52 %)
Grandchild	9 (36 %)
Great grandchild	1 (4 %)
**Person with Dementia Characteristics**	
Gender (number, %)	
Male	8 (32 %)
Female	17 (68 %)
Another identified gender:	0 (0 %)
Prefer not to say	0 (0 %)
Age	
Mean (SD)	81.32 (7.94)
Range	70–96
Ethnicity (number, %)	
Hispanic	2 (8 %)
Non-Hispanic	23 (92 %)
Race (number, %)	
White/Caucasian	12 (48 %)
Black, African American	8 (32 %)
Native American or Alaskan Native	0 (0 %)
Asian	3 (12 %)
Native Hawaiian or Pacific Islander	0 (0 %)
Multi-racial	0 (0 %)
Other	2 (8 %)
Prefer not to say	0 (0 %)

Milestone #2 (≥75 % complete three assigned tasks as demonstration of feasibility). Dashboard data calculating clicks for each task demonstrated that all three tasks were completed by all 25 caregivers (100 % achievement), in addition to all 25 caregivers completing each survey question via Drexel's REDCap program (there were no missing data points).

Milestone #3 (≥75 % rated Plans4Care acceptable). The 25 caregivers rated acceptability using three brief scales described above (AIM, IAM, FIM). We considered this milestone achieved if ≥ 75 % scored between 48 (agree) to 60 (completely agree). We found that 20 (80 %) of the 25 caregivers scored in the designated range.

Milestone #4 (≥75 % rated navigation easy-to-use or ≥68 on SUS, usability). The 25 caregivers rated navigation easy-to-use utilizing the brief 10-item industry standard *Systems Usability Scale*. We found that 96 % of the 25 caregivers scored the platform as easy-to-use (24/25 scored ≥68 which is the industry standard for acceptability of navigation). As per Table 5, the mean SUS score = 86.1. Typically, a score above 70 is considered good, while a score above 85 is considered excellent. Table 6 summarizes the scores obtained for each measure.

**Table 6:** Total scores for outcome measures.

Outcome Measure	Total Sample(N = 25)
**Systems Usability Scale (SUS)**	
Mean (SD)	86.1 (12.23)
Median	85.0
Theoretical Range	0–100
Actual Range	42.5–100
**Acceptability of Intervention Measure (AIM)**	
Mean (SD)	16.28 (3.29)
Median	16
Theoretical Range	4–20
Actual Range	8–20
**Intervention Appropriateness Measure (IAM)**	
Mean (SD)	16.2 (3.08)
Median	16
Theoretical Range	4–20
Actual Range	5–20
**Feasibility of Intervention Measure (FIM)**	
Mean (SD)	16.8 (1.66)
Median	16
Theoretical Range	4–20
Actual Range	14–20

Google Analytics: We also generated Google Analytics to further understand prototype utilization by the 25 caregivers, although these data points did not inform evaluation of milestones. As anticipated, we found that participants in the study spent most of their time on the instruction and Action Plan/strategies pages. Noteworthy is that upon completing the required tasks (including responding to the REDCap survey items), caregivers continued to use the prototype, examined other care challenges, and reviewed their associated strategies generated in the action plans. Most participants (61.5 %) returned to the prototype to generate Action Plans/strategies for other care challenges, and 27 % returned to review the strategies they initially generated. Additionally, 50 % of the time that caregivers were engaged on the prototype was spent reviewing the Action Plan, an important indicator of the salience of the web app and the strategies provided on the Action Plans. These analytics are supported as well by the thematic analyses from the focus groups in which caregivers overwhelmingly reported that the presentation of strategies was extremely helpful.

Qualitative Input from Moderated Evaluation: The themes that emerged supported moving forward with product development and provided important feedback for its future features. Overall, study participants expressed very positive feedback concerning the prototype, praising the sensitivity of the approach and ease of navigation. They appreciated the clear directions provided, and expressed that the prototype was engaging, preserved dignity and independence while ensuring safety. As one participant (female, adult child of person with dementia) explained:

“… I enjoyed the prototype. I thought it was well developed and very sensitive. I found it really easy to navigate … you know, press on a tab, and there's a dropdown. I actually selected difficulty bathing … it just provided such beautiful directives around, …the emphasis on preserving and honoring dignity and independence, but also supporting them and ensuring that they're safe … I like the flow of it. I felt like it was easy to navigate.”

Moreover, some participants wished they had access to the web app early in their caregiving, highlighting its potential usefulness for families at the time of a diagnosis. Some caregivers indicated that even after they completed their tasks for the study, they went back to the prototype to click on other care challenges because the Action Plan was easy to comprehend, and they were eager to obtain additional strategies. As one participant (female, adult child) expressed:

“… I really liked this [Plans4Care web app]. I wish this was available when everything first started in my life, because … I had like no help and I found this app to be amazing. I liked clicking on things, I think even after I did my task I went back and just clicked on some of the other stuff because it just makes so much sense … for someone like me who has no other family around and like no help. It needs almost something to like, guide me in the right direction, and I thought this app was great, and I can't wait to see, like what else we'd be adding to it.”

Overall, participants found the Plans4Care prototype to be practical and helpful.

“… I also found it very practical and easy to implement and digest.” (Male, grandchild)

“… I think this is great, you know. Great knowledge, you know. Knowledge is power.” (Female, grandchild)

Finally, participants expressed that the prototype was “awesome” and they urged for and were excited about its continued development.

“… This is awesome. You guys, thank you for doing this.” (Female, adult child)

“… I really like this. I just think this is really appealing and user friendly.” (Female, adult child)

## 4 Efficacy testing (Phase II)

### 4.1 Overview of study design and aims

**Fig. 3:**
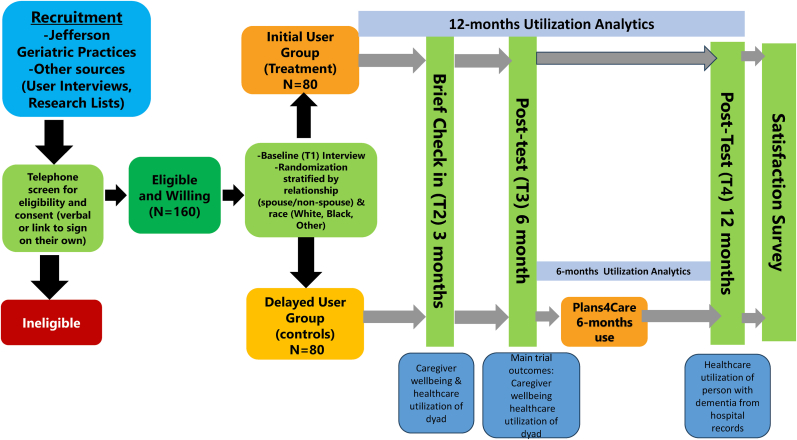
Plans4Care study flow Chart.

The P4C web app will be tested in a prospective randomized controlled trial (NIH stage model III) with 160 caregivers. As shown in Fig. 3, the trial will involve randomizing caregivers to an immediate treatment group who will have access to the app for 12 months or to a wait-list control group who will gain access at 6 months from study entry and be able to use the app for the next 6 months. All study participants will be interviewed by Drexel University research staff at baseline, 3 months for a quick check-in and evaluation of immediate outcomes, 6 months (main study outcomes), and 12 months. The trial will also evaluate utilization patterns.

On an exploratory basis, we will examine the effects of the web app on other important clinical outcomes, including caregiver use of effective communication and simplification strategies. Lastly, study satisfaction and perceived benefits of the web app will be evaluated following the conclusion of a caregiver's study participation.

This design will enable important comparisons and hypotheses to be tested:

1) a comparison of the initial treatment to wait-list control groups at 6 months on caregiver well-being (T1-T2, main trial endpoint)

Hypothesis 1 – Caregivers in the initial treatment vs. wait-list control groups will demonstrate improved wellbeing; between groups)

2) a comparison of the initial treatment group to the wait-list control group at 6 months on healthcare utilization of caregiver and person with dementia (T1-T2, secondary endpoints)

Hypothesis 2 –People with dementia and caregivers in the initial treatment group will have reduced use of certain types of healthcare [fewer 911 calls, fewer emergency department visits, less days hospitalized] at 6 months (between groups, data captured from caregiver report and electronic records);

3) an evaluation of whether the initial treatment group retains 6-month benefits at 12 months (T2-T3)

Hypothesis 3 Caregivers in the initial treatment group will retain improvements in wellbeing from 6 to 12 months (within group analysis

4) an evaluation of whether the wait-list control group achieves similar benefits as the initial treatment group upon receiving access to the platform (T2-T3)

Hypothesis 4 – Wait-list control group caregivers will demonstrate improved wellbeing similar to the initial treatment group (within group analysis)

5) both people with dementia and caregivers in the initial treatment and wait-list control groups will have reduced healthcare utilization at 12 months compared to their baseline 6-month look back (within group analyses, data captured from electronic records).

### 4.2 General procedures

Similar to the eligibility criteria for Phase I prototype testing, study participants (caregivers of individuals with any dementia diagnosis/etiology and disease stage) will be family members, neighbors, or friends who live with or near the person with dementia; identify as their primary caregiver (e.g., person who provides most of the care and support); experience one or more common dementia-related care challenges (e.g. care coordination, managing finances, supporting daily activities of living, managing behavioral symptoms, taking care of themselves, juggling care responsibilities with employment), are ≥21 years of age; are English speaking; have access to a smart phone, tablet, or computer.

Study participants will be recruited from two large primary care practices at Thomas Jefferson University (Center for Healthy Aging and Jefferson Family Medicine Associates) that serve primarily underserved and racially and ethnically diverse individuals.

### 4.3 Recruitment and eligibility

Recruitment strategies within these settings will include sending email blasts and messaging through patient portals. Primary care providers in these practices will also be provided a “prescription” pad with the dedicated study telephone number to offer to their patients/family members. Presentations at staff meetings to inform primary care providers of the research opportunity for their dementia families will be ongoing. Also, IRB approved study flyers will be posted in waiting rooms with QR codes to contact the Drexel research staff to learn about the trial. Caregivers will be contacted directly by the recruiter or encouraged to contact the Drexel University research team through a dedicated study telephone line, email, or by completing a “consent to contact” form located in waiting rooms and also available on the company's website indicating willingness to be contacted to learn about the study. Eligibility criteria are similar to those in Phase I.

### 4.4 Measures

This trial will draw upon three sources of data: caregiver self-report, health utilization data from Jefferson's electronic records, and system/dashboard data capturing use of Plans4Care.

Caregiver self-reported data will be collected by the Drexel research team via a brief telephone screen to determine study eligibility. The screen will include a short questionnaire that operationalizes the eligibility criteria described above. Following determination of eligibility and willingness to participate in the study, verbal or e-consent will be obtained. Then, researchers will administer a 1-h interview to capture basic demographics and baseline data for the main outcome variable (caregiver wellbeing) as well as caregiver appraisal of their health status and health utilization of the person with dementia. As to the latter, this will be a 6-month look back regarding 911 calls, emergency room visits, days hospitalized, and/or short-term nursing home placement for rehabilitative purposes. Subsequent similar brief interviews will occur at 3, 6 (main trial endpoint), and 12 months. At 6- and 12-months, caregivers will also respond to brief questions concerning their endorsement of the platform and satisfaction with its use.

Data will also be captured through the electronic records of Jefferson's primary care practices to track healthcare utilization at 6 and 12 months of the person living with dementia.

Another data source is from the system/dashboard of the online platform, Plans4Care, stored at by Plans4Care, Inc. Dashboard data will include number and date of logins, and number of times using each component of the tool (Resource Library, generation of care plans, monitoring of stress, care challenges addressed, and number of times contacting a care advisor). Table 7 outlines the key measures and the testing occasions in which they will be asked.

**Table 7:** Primary and secondary measures for Phase II randomized trial.

Measure	Description of Measure	Timepoint
Background (Descriptive, co-variate)	Caregivers/people with dementia (gender, race/ethnicity, age, living status, financial strain, employment, education, relationship, time caregiving	T1
Eight item Interview to Differentiate Aging and Dementia (AD8) [46]	Dementia staging as reported by caregiver	T1
Functional Assessment of Staging (FAST) [47]	Dementia staging as reported by caregiver	T1
Caregiver Assessment of Function and Upset (CAFU) [48]	Multidimensional measure of dependence in dementia patients and caregiver reaction	T1, T3, T4
Neuropsychiatric Inventory (NPI) [49,50]	Caregiver report to assess frequency, severity and frequency by severity of neuropsychiatric symptoms in people with dementia	T1, T3, T4
Patient Health Questionnaire (PHQ-8) [51]	Caregiver depressive symptoms	T1, T3, T4
Distress with Behaviors [49]	Caregiver level of distress with behavioral occurrences	T1, T3, T4
Vigilance Scale [52]	Caregiver reports time spent on duty and caring for person with dementia	T1, T3, T4
Perceived Stress (NIH Toolbox Item Bank – PROMIS) [53]	Caregiver perceived level of stress	T1, T3, T4
General Self Efficacy (PROMIS) [54]	Caregiver level of confidence in managing various situations, problems, and events	T1, T3, T4
Perceived Change Index (PCI) [55]	Caregiver well being - assesses caregiver perceived change (gotten worse–a little/a lot, stayed the same, improved-a little/a lot) in affect, somatic, manage daily care.	T1, T2, T3, T4
Negative Communications Scale [[56], [57], [58]]	Used to assess caregiver communication behaviors with people with dementia	T1, T3, T4
Short Sense of Competence Questionnaire (SSCQ) [59]	Overall self-efficacy	T1, T2, T3, T4
Task Management Strategies Index [60]	Strategies caregivers use when providing assistance to a person with dementia	T1, T3, T4
Satisfaction with study and web app use	Brief survey to capture caregiver satisfaction with study and web-app including willingness to pay	T4
Platform use (feasibility, utility)	# of times using platform components (care library, care challenge and care plan; utility of care strategies; # of times care advisor accessed (indicators of fidelity)	ongoing
Resolution of care challenges	Caregivers rate if selected care challenge has been resolved, stayed the same, got better or got worse	ongoing
Health Care Utilization	Caregiver reports 911 calls, emergency room visits, hospitalizations for themselves and person with dementia. Also, caregiver reports nursing home/assisted living and/or adult day service use Also Thomas Jefferson University electronic records from primary care practices of hospitalization, emergency room visits and primary care visits	T1, T2, T3, T4

### 4.5 Power considerations

To calculate sample size for this trial, the research team used these assumptions: a) one primary outcome at 6 months (caregiver wellbeing); b) treatment effect sizes for outcomes from our previous trials (effect sizes for baseline adjusted changes are 0.35 for behavioral symptoms; 0.68 for caregiver distress; and 0.91 for caregiver confidence; c) ability to detect a baseline-adjusted effect at the medium effect size (d) of 0.50 (moderate, nontrivial effect) similar to that observed in our previous trials; d) a type I error rate of 0.05; e) 80 % power; and f) 1:1 randomization.

For the adjusted effect, a correlation between baseline and 6-month scores of at least 0.70 was assumed. Based on these assumptions, 128 caregivers (64 per group) would be needed to attain 80 % power using a standard analysis of covariance with baseline as a covariate for the two treatment groups on the primary endpoint at T3 (6 months). Based on our previous trials, we anticipate a 20 % attrition rate by 6 months (main trial endpoint). Thus, we plan to enroll 160 caregivers.

Although we have not conducted sample size calculations for our secondary outcome (healthcare utilization), there should be sufficient power to support these analyses as they are across the entire study sample.

### 4.6 Randomization scheme

Randomization will occur after completion of the baseline interview. Based on previous trials showing associations between caregiving relationship and race on caregiver outcomes, randomization will be stratified by caregiver relationship (spouse/non-spouse) to people living with dementia and by their race (Black, White, Other) to ensure group balance. Within each relationship-race stratum, a permuted block design will be used to control possible changes over time in participant mix, as well as to eliminate the possibility of study staff predicting the treatment group for the next participant, eliminating selection biases. The randomization schedule will be developed by Plans4Care, Inc.’s biostatistical consultant and will not be disclosed to any other company or research member.

### 4.7 Testing occasions

Following the baseline interview (T1), interviewers will execute the randomization function in RedCap and inform participants of their random assignment (initial user group – treatment group; or delayed user group - controls). If assigned to the immediate user group, the interviewer will provide a link to the Plans4Care platform and onboard the caregiver at that time. As the initial interviewer will be unblinded at this point, another interviewer on the research team will conduct the subsequent follow-up interview at 3-months (T2), which is a brief check-in to keep caregivers connected to the study. If at 3 months the interviewer becomes unblinded, another interviewer will conduct the 6-month interview to maintain blinding (T3, main endpoint). At the conclusion of the 6-month interview, interviewers will be informed of the caregivers’ group; if in the wait-list group, then the interviewer will provide caregivers with a link to the platform and onboarding instructions. All caregivers will be interviewed at 12 months (T4), followed by a brief satisfaction survey.

### 4.8 Data management

Although this is a low-risk trial, this study has a Data and Safety Monitoring Plan and an independent appointed Board (DSMB) to assure participant safety and adherence to human subject protection policies and study protocols. The DSMB will be comprised of 3 members with diverse expertise, including dementia, family caregiving, and digital platforms. Specific responsibilities of the DSMB include: to provide an independent periodic review of recruitment and enrollment progress and adverse events (AEs) including serious events; offer recommendations regarding the trial based on such observed events; and serve in a consultative capacity to the research team regarding study procedures to address ethical dilemmas (e.g., reporting of abuse), safety of study participants in the trial, and appropriateness of all study procedures.

### 4.9 Analytic approach

Data from all caregivers will be part of the primary analyses regardless of actual level of engagement with the web app (intention to treat analysis, ITT), so that each caregiver will be included in the analysis in the group to which they are randomized. We will use analysis of covariance (ANCOVA) to assess differences at each endpoint, with baseline level of the outcome variable serving as covariate. Treatment and control groups will be balanced on caregiving relationship and caregiver race due to our stratified randomization procedure, but we will examine other demographic and baseline variables (e.g., age, gender) and if random imbalances are found on these potential confounders between the immediate treatment and wait-list control groups, we will adjust for them in our models by including them as additional covariates. Should the degree of missing data be large, we will conduct multiple imputation models in which missingness is modeled as a function of covariates, to impute missing outcomes. This will serve as a sensitivity analysis to the main analysis of complete data only. We will also examine the consistency of the treatment effects across the multiple primary care practices in additional sensitivity analyses, although we assume that the intervention effect will be consistent and that pooling the data across practices will be appropriate.

To examine if the wait-list control group demonstrates similar benefits upon gaining access to the P4C web app at 6 months, we will examine within group changes from T3 to T4 using appropriate within-group pairwise comparisons and then compare them to the magnitude of change in the immediate treatment group (T1-T3). We will also examine system data to determine the number and type of care challenges selected and Action Plans generated from baseline to 6 months (T1-T3) for the initial user group and from 6 to 12 months (T3-T4) for both groups. Analyses of predictors of platform engagement are important to identify which caregivers are more likely to use the platform and to help modify the system for those who may not use it. We will examine frequency plots and specify thresholds of platform engagement, then use linear regression models to identify predictors of engagement from baseline to 6 and 12 months.

### 4.10 Initial user group

Following the baseline interview, caregivers assigned to the initial user group will receive access to the web app and an orientation to the web app involving initial creation of an account and completing a brief set of background questions.

### 4.11 Delayed user group (wait-list control group)

Participants randomized to the delayed user group will receive access to the web app at the conclusion of their 6-month interview. Similar procedures as above will be followed to onboard caregivers to the web app.

At 12 months, access will be closed by account deactivation for all participants, and they will be instructed how to remove the app from their devices. They will receive a book that has many of the strategies on the P4C web app [36].

### 4.12 Data collection

Data will be collected via telephone at each testing occasion: screen, baseline, 3, 6, and 12-month interviews. Caregivers can choose to complete an interview in one phone interview (up to 90 min) or in segments over a two-week timeframe. The baseline interview involves self-reported data about the caregiver's health and wellbeing, use of communication, and simplification strategies. Caregiver-reported information on the person with dementia's health, daily function, and care needs will also be collected. Follow-up interviews at 3, 6, and 12 months collect primary outcome data.

Additionally, the web app dashboard will capture the frequency of caregiver logins and use of its five components. Finally, following study completion, a study team member other than the interviewer will conduct a 15-min telephone survey to assess the caregiver's experience as a study participant and using the P4C web app.

Exploratory analyses will evaluate the impact of web app use on other outcomes of interest. Possible intervention moderator effects by caregiver gender, race, and relationship (spouse/non-spouse) will also be examined. Mediation modeling will be conducted using structural equation modeling techniques to determine what proportion of the web app's impact on primary outcomes can be explained by its impact on change in theoretically derived mediating variables (self-efficacy, competence). Methods for testing intervention effects in the context of randomized trials with multiple waves of mediator and outcome data will be implemented.

## 5 Discussion

The Plans4Care web app addresses a critical public health priority to support family caregivers of people living with dementia. Its novel features address the limitations of current mobile apps on the market and include the expansive number of care challenges addressed, the assessment of cognitive functional capacity of the person with dementia, strategies that span the spectrum of cognitive function, education materials, strategies that are relevant to any disease stage and etiology, the personalization of strategies, and access to a trained care advisor as needed. The P4C web app is grounded in a conceptual model for understanding caregiver needs and is the first to operationalize an algorithmic approach to map nonpharmacological strategies to cognitive functional capacity and other contextual factors. It does not require caregivers—who are typically pressed for time—to complete training sessions over the course of weeks or months, as in individual or group interventions to learn dementia care strategies [15]. Additionally, caregivers can navigate through any of the components of the P4C web app based on their preference, needs, and time, enabling self-direction, placing caregivers in control and independent of health professional to address common daily challenges. Thus, its cost would be minimal and its potential for dissemination, scalability, and reach are high. Family caregivers have limited access to strategies that are personalized to their unique situations and yet they face increasing challenges with disease progression. The P4C web app has potential to improve caregiver wellbeing, support care decision-making across disease progression, and improve quality of care and safety at home.

Each of its components and approach to engagement is grounded in evidence. As such, P4C web app offers an innovative, nonpharmacological option that health providers in any setting (homecare, hospital, rehabilitation, primary care, Medicaid Waiver, Managed care) can provide families. Health providers themselves can use the P4C web app and/or serve as care advisors with minimal orientation needed to enable them to deliver vetted disease education and science-backed strategies specific to the circumstances of caregivers they are serving.

Evidence suggests that providing knowledge and skills to caregivers via web-based dynamic platforms can be effective [21,22,37]. By enabling families to access the P4C web app via smart phone, tablet, or computer technology may also address the persistent disparity in access to broadband internet among various populations, including rural families with limited resources but who have smart phones [38,39].

Future iterations of Plans4Care will integrate AI-type features, voice activation of strategies and guides, and use of language learning technologies to analyze interactions with care advisors from which to generate session summations, strategies discussed, and action steps.

Web app developers confront similar challenges, and that is determining best route for commercialization, reaching those most in need but who may have limited experience with technologies, and identifying price structures for consumers and/or healthcare organizations.

In conclusion, lessons learned from this trial that will evaluate efficacy and app utilization may shorten the time frame of the classic 17-plus year gap from idea inception to widespread use of evidence [40] and set the stage for dissemination, scaling, and commercialization opportunities.

## CRediT authorship contribution statement

**Laura N. Gitlin:** Writing – review & editing, Writing – original draft, Methodology, Investigation, Funding acquisition, Formal analysis, Data curation, Conceptualization. **Eric Jutkowitz:** Writing – review & editing, Validation, Methodology, Investigation, Funding acquisition, Conceptualization. **Catherine Piersol:** Writing – review & editing, Validation, Methodology, Investigation, Funding acquisition, Conceptualization. **Sokha Koeuth:** Writing – review & editing, Validation, Project administration, Formal analysis, Data curation. **Taylor Sivori:** Writing – review & editing, Project administration, Formal analysis, Data curation. **Melinda J. Webster:** Writing – review & editing, Project administration, Formal analysis, Data curation. **Rachel N. Barnett:** Writing – review & editing, Data curation. **David L. Roth:** Writing – review & editing, Methodology, Formal analysis.

## Ethics and consent statement

Drexel University's IRB served as the single IRB of record. We will seek verbal consent from participating caregivers. The consenting process will be overseen by the Drexel research staff using an IRB approved assenting process. At the telephone screen, we will seek verbal assent from family caregivers to participate in the screen. Family caregivers will be informed that their participation in the telephone interviews is confidential and completely voluntary. The objectives, procedures, and a clear statement (IRB approved) explaining risks and benefits of this study will be presented at the telephone screen. For those eligible based on the telephone screen and who agree to participate, an appointment will be scheduled for the baseline interview or conducted following the screen. Prior to conducting the baseline interview by telephone, the interviewer will again review all study procedures and seek verbal or e-consent which will be documented in REDCap. Family caregivers will be sent a PDF copy of the consent as an email attachment for their records. Dr. Gitlin and her team have used this approach effectively in another trial testing a different online platform.

## Compliance to ethical standards

All work has or will be conducted in compliance with confidentiality (HIPAA) standards.

## Adherence with ethical standards

This trial protocol has been approved by Drexel University's IRB #2306009970. Clinical Trial Registry #: 2306009970.

## Funding source

National Institute on Aging (Small Business Initiative Research Fast Track) Grant #4R44AG084365-03.

## Declaration of competing interest

The authors declare the following financial interests/personal relationships which may be considered as potential competing interests: Laura Nan Gitlin reports was provided by Drexel University. Laura Nan Gitlin reports a relationship with Plans4Care, Inc. that includes: board membership. Laura Nan Gitlin has patent #410259–501P01US pending to Laura N. Gitlin, Eric Jutkowitz, Catherine V. Piersol. If there are other authors, they declare that they have no known competing financial interests or personal relationships that could have appeared to influence the work reported in this paper.

## Data Availability

Data will be made available on request.
